# MicroRNAs: new biomarkers and promising therapeutic targets for diabetic kidney disease

**DOI:** 10.1590/2175-8239-JBN-2018-0165

**Published:** 2019-02-07

**Authors:** Linicene Rosa do Nascimento, Caroline Pereira Domingueti

**Affiliations:** 1 Universidade Federal de São João Del Rei DivinópolisMG Brasil Universidade Federal de São João Del Rei, Campus Centro-Oeste Dona Lindu, Divinópolis, MG, Brasil.

**Keywords:** Diabetic Nephropathies, MicroRNAs, Drug Therapy, Biomarkers, Early Diagnosis, Prognosis

## Abstract

Diabetic kidney disease (DKD) is a chronic complication of diabetes *mellitus* associated with significant morbidity and mortality regarded as a global health issue. MicroRNAs - small RNA molecules responsible for the post-transcriptional regulation of gene expression by degradation of messenger RNA or translational repression of protein synthesis - rank among the factors linked to the development and progression of DKD. This study aimed to offer a narrative review on investigations around the use of microRNAs in the diagnosis, monitoring, and treatment of DKD. Various microRNAs are involved in the pathogenesis of DKD, while others have a role in nephroprotection and thus serve as promising therapeutic targets for DKD. Serum and urine microRNAs levels have also been considered in the early diagnosis and monitoring of individuals with DKD, since increases in albuminuria, decreases in the glomerular filtration rate, and progression of DKD have been linked to changes in the levels of some microRNAs.

## Introduction

Diabetes *mellitus* (DM) has been associated with numerous debilitating conditions including diabetic kidney disease (DKD), one of the main reasons for prescribing dialysis to individuals with DM.^1^ DKD has become one of the main causes of kidney failure and a prominent global health issue. It has been described as one of the main causes of death of diabetic patients.^2^

Early diagnosis of DKD may prevent the progression of renal disease and the onset of cardiovascular events.^3^ New markers are required to assess renal function, since glomerular filtration rate (GFR) and urinary albumin excretion (UAE) have limited use in detecting early-stage DKD.^4^

Promising markers include neutrophil gelatinase-associated lipocalin (NGAL), N-Acetyl-β-D-Glucosaminidase (NAG), kidney injury molecule-1 (KIM-1), α1- and β2-microglobulin, liver-type fatty acid binding protein (L-FABP), and retinol binding protein (RBP4).^3^ Some of these markers may be detected when the UAE increases and the GFR decreases.^5^ MicroRNAs have been regarded as promising markers for the early diagnosis and monitoring of DKD.^6^

MicroRNAs are small non-coding RNA molecules containing about 22 nucleotides. They are responsible for the post-transcriptional regulation of gene expression by degradation of messenger RNA or translational repression of protein synthesis.^7^ MicroRNAs have been regarded as powerful regulators of numerous conditions that may critically impact the onset and/or progression of diseases such as DKD.^8^^,^^9^ This study aimed to offer a narrative literature review on the role of microRNAs in the diagnosis, monitoring, and treatment of DKD.

## Material and methods

Searches were carried out on databases Medline/PubMed and SciELO for papers looking into the use of serum or urine levels of microRNAs in the diagnosis and monitoring of individuals with DKD and studies performed with animal models or cell cultures to assess microRNAs as potential therapeutic targets for DKD.

### Diabetic kidney disease

DM involves a number of metabolic disorders having hyperglycemia as a common thread. Chronic hyperglycemia may cause injury to the capillaries of the glomeruli and result in chronic kidney disease (CKD).^10^ CKD has been defined as the presence of anomalous kidney function or renal structures lasting for more than three months that cause harm to one's health.^6^ DKD is CKD occurring in a progressive fashion, an asymptomatic condition that progresses with the loss of renal function and requires the prescription of dialysis and even kidney transplantation to individuals with more advanced stages of the disease. It decreases patient quality of life and increases the risk of early death, particularly for cardiovascular causes, regardless of the level of renal involvement.^3^

DKD is one of the main complications of diabetes *mellitus* types 1 (DM1) and 2 (DM2). Classic histology findings include mesangial expansion, mesangial hypertrophy, reduced podocyte number, and protein accumulation in the extracellular matrix, glomeruli, and tubular compartments, including collagen, a protein associated with fibrosis. The main signs of the disease are albuminuria and glomerular proteinuria. DKD is found in 20-40% of the individuals with DM and ranks as the main cause of end-stage renal disease.^11^

Screening for DKD must commence as soon as patients are diagnosed with DM2 and five years after a diagnosis of DM1, unless the individual with DM1 is in puberty or presents with uncontrolled hyperglycemia. In this case, screening tests must be performed earlier. Screening must be carried out annually based on UAE and GFR testing.^3^

The criteria used to diagnose individuals with DKD are GFR below 60 mL/min/1.73m^2^ and/or increased UAE for at least three months. Increased UAE is defined as an albumin-to-creatinine ratio (ACR) ≥ 30 mg/g or albumin levels ≥ 30 mg in 24-hour urinary protein. The simultaneous assessment of GFR and UAE allows for early diagnosis and enables the categorization of CKD (Chart 1) and the subsequent prognosis and therapeutic measures applicable to each stage of the disease.^12^

**Chart 1:** Stages of diabetic kidney disease based on the glomerular filtration rate and urinary albumin excretion

Stage	Description	Glomerular filtration rate (mL/min/1.73m^2^)
G1	Normal and high GFR*	≥ 90
G2	Mild GFR reduction*	60-89
G3a	Mild to moderate GFR reduction	43-59
G3b	Moderate to severe GFR reduction	30-44
G4	Severe GFR reduction	15-29
G5	Kidney failure	< 15
Stage	Description	Albumin urinary excretion (mg/g of creatinine or mg/24 hours)
A1	Normal albuminuria	< 30
A2	Moderate to severe albuminuria	≥ 30 e < 300
A3	Severe albuminuria	≥ 300

* In the absence of markers of renal parenchymal injury, stages G1 and G2 are not used for the diagnosis of CKD.

Adapted from KDIGO^11^

The treatment of DKD is currently designed to decrease UAE, prevent the progression of the disease, reduce the rate at which the GFR decreases, and prevent cardiovascular events.^3^ Proper disease control requires optimized management of blood sugar levels, maintenance of glycosylated hemoglobin (HbA1c) levels below 7.0%, and well-controlled blood pressure levels (≤ 140/90 mmHg if UAE < 30 mg/24h and ≤ 130/80 if UAE ≥ 30 mg/24h) to mitigate the risk and decelerate the progression of DKD.^10^^,^^11^

DKD combined with dyslipidemia increases preexisting cardiovascular risk and further increases the risk of death by cardiovascular disease of individuals with DM1 or DM2. Lipid-lowering drugs are recommended for patients with DM with or without CKD aged 40+ years with one or more cardiovascular risk factors such as LDL cholesterol levels ≥ 100 mg/dL, high blood pressure, smoking, overweight or obesity, and previous diagnosis of coronary artery disease.^11^

Nephroprotective drugs also play an important role in preventing the progression of DKD. Renin-angiotensin-aldosterone system inhibitors have well-established positive effects in the preservation of the GFR and in the reduction of albuminuria. Nephroprotective mechanisms combine to improve glomerular hemodynamics, restore the function of the glomerular filtration barrier, and limit effects of angiotensin II and aldosterone such as fibrosis and vascular endothelial dysfunction. Angiotensin-converting-enzyme (ACE) inhibitors and angiotensin II receptor blockers (ARBs) are the drugs of choice to manage the blood pressure of patients with DM while preventing risk and impeding the progression of DKD.^12^

Few nephroprotective drugs are currently available for the treatment of individuals with DKD. New therapeutic targets must be sought to foster the development of more effective medication.^3^ Some authors have shown that different microRNAs are involved in the pathogenesis of DKD, which makes them interesting therapeutic targets.^13^^,^^14^

### MicroRNAs

MicroRNAs were discovered about 20 years ago and their involvement in several biological processes and in the pathogenesis of numerous diseases has been studied since.^13^ Although microRNAs have been known for quite a while, knowledge of their function and mechanisms of action is still limited. The human genome contains more than 1000 microRNAs, and estimates indicate that some 60% of the human protein-coding genes may be regulated by microRNAs, which means they may significantly affect the expression of a number of proteins.^15^

MicroRNAs are small molecules containing about 22 nucleotides produced inside cells as short regulating non-coding RNA. They regulate a number of fundamental biological pathways and act upon various cell functions^15^ to induce normal and pathological conditions in myriad biological systems.^14^ They may be found in animals, plants, and some viruses, and act on RNA silencing and regulate post-transcriptional gene expression.^16^

MicroRNAs are usually found in the intracellular milieu, although circulating microRNAs may also be present in the extracellular environment.^17^ MicroRNAs decrease target gene expression and consequently alter cell proliferation, apoptosis, and differentiation during the evolution of mammals.^18^ They silence target genes by binding to 3'UTR during transcription and repressing target messenger RNA or promoting the degradation of messenger RNA by cleavage.^19^ Interestingly, microRNAs are heterogeneous, i.e., they may bind to various messenger RNAs to simultaneously silence multiple genes.^20^

Minor changes in microRNA levels may produce significant cell-related effects. Changes in the expression of microRNAs may also be observed in the development of many human diseases.^14^ A recent study looked into the effects of bariatric surgery on serum microRNA levels in individuals with DM2. The expression levels of microRNA29a-3p, microRNA122-5p, microRNA124-3p, and microRNA320a, all of which associated with DM2, decreased after bariatric surgery. MicroRNA expression levels changed after bariatric surgery and promoted glucose-induced insulin secretion, decreased insulin resistance, and protected beta cell function.^21^ These outcomes suggest that changes to microRNA expression levels occurring in obese individuals might be related to the development of DM2.

Some microRNAs found in the kidneys were deemed essential for good renal function. Changes in the expression of these microRNAs might significantly contribute to the development of renal diseases such as DKD, acute kidney injury, lupus nephritis, polycystic kidney disease and others, since they affect the genes involved in the pathogenesis of these conditions. Therefore, they are potentially good therapeutic targets for these renal diseases.^14^

MicroRNAs are also potential prognostic markers for different renal diseases, since they are considerably stable and are present in various biological materials. The development of new diagnostic and therapeutic techniques involving microRNAs for future use in the diagnosis, treatment, and prevention of kidney diseases holds promise.^14^

MicroRNAs are promising early diagnostic and DKD monitoring markers on account of their stability in urine and blood. Some are specifically linked to DKD. The urine and serum of individuals with DKD contain sediments with microRNAs that may correlate with specific stages of kidney disease, fibrosis, and renal function decrease. Exosomes in urine are an excellent tool for the analysis of microRNAs in renal diseases, since many originate in kidney cells.^15^

MicroRNAs have gained strength as renal biomarkers and offered good perspectives for the future clinical management of DKD as an addition to GFR and albuminuria testing in disease diagnosis and monitoring.^22^

### Promising uses of microRNAs in the diagnosis and monitoring of diabetic kidney disease

MicroRNAs are stable and may be detected in human fluids. The detection of microRNA in biological materials is relevant in clinical research for the development of diagnostic biomarkers for DKD, since early diagnosis may prevent progression to kidney failure and cardiovascular events.^15^

AlKafaji^23^ recently described an association between the expression of microRNA-126, DM2, and DKD. The study included 52 patients with DM2 and normal albuminuria, 50 patients with DM2 and increased albuminuria (29 with moderate to severe and 21 with severe albuminuria), and 50 non-diabetic healthy individuals. Expression of microRNA-126 was significantly lower in individuals with DM2 and even lower in patients with DKD when compared to controls. MicroRNA-126 levels were also correlated with albuminuria, with significantly lower expression in individuals with moderate to severe albuminuria and even lower expression in patients with severe albuminuria in relation to patients with DM2 and normal albuminuria. MicroRNA-126 levels were negatively correlated with albuminuria and positively correlated with the GFR. Therefore, these findings indicated that decreased expression of circulating microRNA-126 might be related to the development of DKD in individuals with DM2, suggesting that microRNA-126 might be nephroprotective and a promising biomarker for the diagnosis and monitoring of DKD.

A longitudinal study conducted by Argyropoulos^24^ looked into urine microRNA profiles of patients with DM1 and DKD. The study included 40 patients with DM1 followed for 20 years - ten without DKD, ten with severe albuminuria, ten with intermittent moderate to severe albuminuria, and ten with persistent moderate to severe albuminuria. At the start of the study, the patients with persistent moderate to severe albuminuria had decreased levels of microRNA-323b-5p and increased levels of microRNA-122-5p and microRNA-429 in relation to individuals with intermittent moderate to severe albuminuria. The onset of moderate to severe albuminuria was associated with decreased levels of microRNA-323b-5p and increased levels of microRNA-429. Nine microRNAs (microRNA-619; microRNA-486-3p; microRNA-335-5p; microRNA-552; microRNA-1912; microRNA-1224-3p; microRNA-424-5p; microRNA-141-3p; microRNA-29b-1-5p) had increased urine expression levels in individuals with moderate to severe albuminuria, whereas microRNA-221-3p had decreased expression. Therefore, the study showed that microRNA urinary profiles diverged between individuals with DKD of different stages, thus showing their use as markers for the diagnosis and risk stratification of DKD of patients with DM1.

In another more recent longitudinal study, Argyropoulos^25^ assessed the expression of 723 microRNAs in the urine of 27 patients with DM1 and normal albuminuria, ten without DKD and 17 with moderate to severe albuminuria. Eighteen microRNAs were significantly associated with further development of moderate to severe albuminuria, indicating that this change in microRNA levels might be useful in predicting the development of DKD and in the early diagnosis of the condition. Conversely, microRNA-10, microRNA-23, microRNA-30, and microRNA-200 were among the microRNAs with higher expression levels in the urine of the group without DKD, suggesting a possible nephroprotective effect.

MicroRNA expression in urinary exosomes was evaluated in a study carried out by Barutta^26^ with 12 patients with DM1 and normal albuminuria and 12 individuals with DM1 and moderate to severe albuminuria. Higher levels of microRNA-130a and microRNA-145 and lower levels of microRNA-155 and microRNA-424 were observed in urinary exosomes of individuals with moderate to severe albuminuria when compared to subjects with normal albuminuria. The changes in the levels of microRNAs in the urinary exosomes of patients with DM1 and DKD indicate they might be good biomarkers for DKD.

Barutta^27^ analyzed the serum levels of 377 microRNAs in a cross-sectional study enrolling 455 patients with DM1. Patients with one or more complications stemmed from DM added up to 312, and 143 subjects did not have evidences of complications from DM. Patients with one or more complications from DM had altered expression levels in 25 microRNAs. MicroRNA-126 levels analyzed by qRT-PCR were significantly lower in individuals with increased albuminuria (n = 179) when compared to controls, indicating a beneficial renal effect of microRNA-126 in patients with DM1 and a potential clinical use of measuring the level of this microRNA in the diagnosis of DKD.

A longitudinal study assessed the expression of 13 microRNAs of 14 patients with DM1 and DKD before and after pancreas and kidney transplantation. The authors reported that microRNA expression became normal after transplantation, indicating they may have an effect in the pathogenesis of DKD and serve as biomarkers of this complication of DM. A cross-sectional analysis of the data performed in this study compared patients with DM1 and GFR < 30 mL/min (n = 21) to subjects with DM1 and GFR ≥ 30 mL/min (n = 15). The individuals with GFR < 30 mL/min had higher expression levels of microRNA181a and microRNA-326 and lower expression levels of microRNA-126 and microRNA-574-3p when compared to the subjects with GFR ≥ 30 mL/min, revealing a change in the expression of these microRNAs in the more advanced stages of DKD, thus indicating a possible clinical use for these microRNAs at monitoring the progression of DKD.^28^

Wang et al.^29^ analyzed the serum microRNA expression levels of 184 patients with DM2 - 92 with microvascular complications and 92 without complications - matched for age and sex. Five microRNAs were significantly more expressed in the individuals with DM2 with microvascular complications. These five microRNAs were positively correlated with serum glucose and triglyceride levels and negatively correlated with serum high-density lipoprotein (HDL) cholesterol levels. These findings suggest that the positive regulation of these five microRNAs in individuals with DM2 might be involved in the pathogenesis of DM2 and diabetic microvascular complications.

El-Samahy et al.^30^ studied microRNA-377 and microRNA-216a as biomarkers of DKD and risk of atherosclerosis in children with DM1 versus controls. The results showed that microRNA-377 levels in urine were significantly higher, while microRNA-216a levels were significantly lower in patients with increased albuminuria (n = 24) compared to patients with normal albuminuria (n = 26). The detection of moderate to severe albuminuria in patients with DM1 through urinary microRNA-377 achieved a sensitivity of 92% and a specificity of 85%, whereas for microRNA-216 sensitivity was 91.3% and specificity 84,1%. Significant positive correlations were found between urinary microRNA-377 and HbA1c, ACR, carotid intima-media thickness, while urinary microRNA-216a was negatively correlated with these variables. Therefore, urinary microRNA-377 and microRNA-216a may be deemed as early biomarkers for kidney disease and subclinical atherosclerosis in patients with DM1.

Table 1 summarizes the main results of studies on the use of microRNAs in DKD diagnosis and monitoring. Table 2 lists the microRNAs with lower or higher expression levels in individuals with DKD. The list of microRNAs with higher and lower expression levels in individuals with DKD varied broadly among studies. MicroRNA-126 was the only to present lower expression levels in three studies with individuals with DKD. A meta-analysis^31^ also reported significantly decreased serum microRNA-126 and significantly increased microRNA-770 levels in patients with DKD.

**Table 1:** Studies assessing the use of microRNAs in the diagnosis and monitoring of diabetic kidney disease

Author/Year	Patient categorization	Sample type	Study design	Result
Al Kafaji *et al*., 2016^23^	52 with DM2 & normal albuminuria 29 with DM2 & moderately increased albuminuria 21 with DM2 & severely increased albuminuria	Serum	Cross-sectional	MicroRNA-126 expression levels were lower in patients with moderately increased albuminuria and even lower in patients with severely increased albuminuria in relation to subjects with normal albuminuria.
Argyropoulos *et al*., 2013^24^	10 with DM1 without DKD 10 with DM1 & severely increased albuminuria 10 with DM1 & intermittent moderately increased albuminuria 10 with DM1 & persistent moderately increased albuminuria	Urine	Longitudinal	MicroRNA-323b-5p levels decreased and microRNA-429 levels increased in patients with moderately increased albuminuria, while the levels of microRNA-619; microRNA-486-3p; microRNA-335-5p; microRNA-552; microRNA-1912; microRNA-1224-3p; microRNA-424-5p; microRNA-141-3p; microRNA-29b-1-5p increased and the levels of microRNA-221-3p decreased in patients with severely increased albuminuria.
Argyropoulos *et al*., 2015^25^	27 with DM1 & normal albuminuria (10 did not develop DKD & 17 developed moderately increased albuminuria)	Urine	Longitudinal	Increased expression levels of microRNA-495; microRNA-548o-3p; microRNA-7a-5p; microRNA-1247-5p; microRNA-767-3p; microRNA-122-5p; microRNA-645; microRNA-199a-5p; microRNA-7b-3p; microRNA-30a-5p; microRNA-17-5p; microRNA-126-3p; microRNA-548c-3p; microRNA-665; microRNA-640; microRNA-302a-3p; microRNA-616-5p; microRNA-770-5p was associated with moderately increased albuminuria, while expression of microRNA-10; microRNA-23; microRNA-30; microRNA-200 was associated with non-development of DKD.
Barutta *et al*., 2013^26^	12 with DM1 & normal albuminuria 12 with DM1 & moderately increased albuminuria	Urine	Cross-sectional	Expression levels of microRNA-130a & microRNA-145 were higher and expression levels of microRNA-155 & microRNA-424 were lower in patients with moderately increased albuminuria in relation to normal albuminuria.
Barutta *et al*., 2016^27^	179 with DM1 & increased albuminuria 143 with DM1 and without DKD	Serum	Cross-sectional	MicroRNA-126 levels were lower in patients with increased albuminuria in relation to controls.
Bijkerk *et al*., 2015^28^	21 with DM1 & GFR < 30 mL/min 15 with DM1 & GFR ≥ 30 mL/min	Serum	Cross-sectional	MicroRNA181a & microRNA-326 levels were increased and microRNA-126 & microRNA-574-3p levels were decreased in patients with GFR < 30 mL/min in relation to patients with GFR ≥ 30 mL/min.
Wang *et al*., 2016^29^	92 with DM2 & microvascular complications 92 with DM2 without complications	Serum	Cross-sectional	The levels of microRNA-661; microRNA-571; microRNA-770-5p; microRNA-892b; microRNA-1303 were increased in patients with microvascular complications in relation to patients without complications.
El-Samahy et al., 2018^30^	26 with DM1 & normal albuminuria 24 with DM1 & increased albuminuria	Urine	Cross-sectional	MicroRNA-377 levels were increased and microRNA-216a levels were decreased in patients with increased albuminuria in relation to patients with normal albuminuria.

DM1 = diabetes *mellitus* type 1; DM2 = diabetes *mellitus* type 2; DKD = diabetic kidney disease; GFR = glomerular filtration rate.

**Table 2:** MicroRNAs with decreased or increased expression levels in patients with diabetic kidney disease

MicroRNAs with decreased expression levels	Reference
microRNA-126	Al Kafaji et al., 2016^23^
microRNA-221-3p; microRNA-323b-5p	Argyropoulos et al., 2013^24^
microRNA-10; microRNA-23; microRNA-30; microRNA-200	Argyropoulos et al., 2015^25^
microRNA-155; microRNA-424	Barutta et al., 2013^26^
microRNA-126	Barutta et al., 2016^27^
microRNA-126; microRNA-574-3p	Bijkerk et al., 2015^28^
microRNA-216a	El-Samahy et al., 2018^30^
MicroRNAs with increased expression levels	Reference
microRNA-29b-1-5p; microRNA-141-3p; microRNA-335-5p; microRNA-424-5p; microRNA-429; microRNA-486-3p; microRNA-552; microRNA-619; microRNA-1224-3p; microRNA-1912	Argyropoulos et al., 2013^24^
microRNA-7a-5p; microRNA-7b-3p; microRNA-17-5p; microRNA-30a-5p; microRNA-122-5p; microRNA-126-3p; microRNA-199a-5p; microRNA-302a-3p; microRNA-495; microRNA-548c-3p; microRNA-548o-3p; microRNA-616-5p; microRNA-640; microRNA-645; microRNA-665; microRNA-767-3p; microRNA-770-5p; microRNA-1247-5p	Argyropoulos et al., 2015^25^
microRNA-130a; microRNA-145	Barutta et al., 2013^26^
microRNA181a; microRNA-326	Bijkerk et al., 2015^28^
microRNA-571; microRNA-661; microRNA-770-5p; microRNA-892b; microRNA-1303	Wang et al., 2016^29^
microRNA-377	El-Samahy et al., 2018^30^

In a literature review, Yang et al.^32^ found increased levels of microRNA-377, microRNA-192, microRNA-216/217, and microRNA-144, and decreased levels of microRNA-21 and microRNA-375 in the bodily fluids of patients with DKD. The authors also observed that despite the occurrence of significant differences in the urinary excretion of microRNAs in patients with DKD, they were generally not correlated with serum microRNA levels, indicating that urinary microRNA was a better diagnostic marker of DKD.

A systematic review^33^ reported that microRNA-21-5p, microRNA-29a-3p, microRNA-126-3p, microRNA-192-5p, microRNA-214-3p, and microRNA-342-3p were involved in the pathogenesis of DKD and were potential biomarkers for the disease. A meta-analysis^34^ showed higher expression levels of microRNA-21-5p, microRNA-146a-5p, and microRNA-10a-5p, while microRNA-25-3p and microRNA-26a-5p had lower expression levels in individuals with DKD. Another meta-analysis^35^ described higher expression levels of microRNA-142-3p, microRNA-223-3p, microRNA-21-5p, microRNA-142-5p, and microRNA-214-3p and lower expression levels of microRNA-29c-3p and microRNA-200a-3p in subjects with renal fibrosis.

### MicroRNAs as therapeutic targets for diabetic kidney disease

Kang and Xu^36^ described atrasentan, a selective endothelin A receptor antagonist, as a promising drug in the treatment of DKD. The authors noted that atrasentan decreased the expression of microRNA-199b-5p and increased klotho levels, an anti-aging, single-pass protein that controls sensitivity to insulin. Elevation of serum klotho levels mediated by microRNA-199b-5p is a possible mechanism by which atrasentan prevents renal tubular injury in individuals with DKD.

Renin-angiotensin-aldosterone system inhibitors help decrease intraglomerular pressure and hyperfiltration, and are known to decrease proteinuria in patients with DM1 or DM2.^37^ Zhu^38^ reported that losartan inhibited the expression of microRNA-503 and microRNA-181d in the glomeruli of rats, which improved from DKD and had perceptible improvements in albuminuria and kidney injury. This study indicated that the nephroprotective effect provided by losartan included the increased expression of some microRNAs, which by their turn are important therapeutic targets for DKD.

Bai^39^ et al. showed that microRNA-130b levels were significantly decreased in patients with DKD, and that they were negatively correlated with serum creatinine, β2 microglobulin, and proteinuria. The authors also saw that repressing microRNA-130b increased Snail expression in cell cultures exposed to high glucose concentrations. Increased Snail expression has been associated with increased expression of collagen IV, which may contribute to the onset of interstitial fibrosis in individuals with DKD. Therefore, microRNA-130b is a very promising target for the treatment of DKD.

Many studies have described associations between microRNAs and inflammatory markers of DKD, some of which are cited below. Guo et al.^40^ observed that microRNA-29 stimulates the expression of interleukin 6 (IL6) and tumor necrosis factor alpha (TNFα) in the glomeruli of rats. Shao et al.^41^ found that microRNA-217 induces the production of transforming growth factor-β (TGF-β), endothelin, and fibronectin in the glomerular mesangial cells of rats, resulting in renal fibrosis. MicroRNA-192 has also been associated with increased expression of TGF-β,^1^ while microRNA-26a, microRNA-30c, and microRNA-93 have been associated with decreased expression of TGF-β^2^^,^^42^ in the mesangial cells of rats, thus mitigating renal fibrosis and offering a nephroprotective effect.

Wu et al.^43^ found that exposure to high glucose levels increased the expression of microRNA-27a in glomerular mesangial cell cultures of diabetic rats. MicroRNA-27a inhibition with the administration of antagonist drugs resulted in lower proliferation of mesangial cells induced by high glucose levels, lower expression of profibrotic genes associated with the extracellular matrix, and decreased renal fibrosis and renal hypertrophy in mice, indicating that microRNA-27a antagonists are promising candidates for the treatment of DKD.

Sitagliptin, a medication used in the treatment of DM2, is a promising agent for the treatment of DKD on account of the improvements described in microRNA-200a-mediated oxidative stress in rats with DKD.^44^ Xu et al.^45^ also reported that resveratrol induces the expression of microRNA18a-5p in rat podocytes with subsequent improvements in DKD, an indication that stimulation of this particular microRNA might be a promising therapy for DKD.

Kolling et al.^8^ reported that microRNA-21 is among the most expressed microRNAs in the kidneys of mice with DKD and that in vitro and in vivo inhibition of this microRNA decreased mesangial matrix expansion, interstitial fibrosis, macrophage infiltration, podocyte loss, da albuminuria, and the expression of inflammatory and fibrotic molecules. MicroRNA-21 antagonists may improve the structural and functional parameters of kidneys of mice with DKD, and are thus promising agents to treat this complication in subjects with diabetes.

Han et al.^46^ looked into the effects of triptolide in the treatment of microRNA-137-mediated DKD. Although significantly decreased in cells exposed to high glucose levels and in the kidney tissue of diabetic rats, microRNA-137 expression was induced by triptolide. Increased microRNA-137 expression and triptolide had similar effects, while microRNA-137 inhibition intensified the accumulation of proteins in the extracellular matrix. MicroRNA-137-dependent effects were associated with increased NOTCH1 expression, which in turn inhibits the expression of proteins in the extracellular matrix, important mediators of glomerulosclerosis.

**Figure 1:**
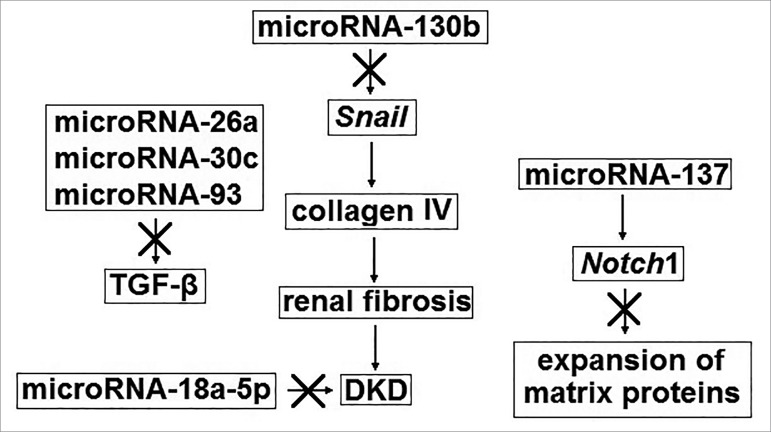
Mechanism of action of microRNAs with possible nephroprotective properties. DKD = diabetic kidney disease; TGF-β = transforming growth factor beta.

**Figure 2:**
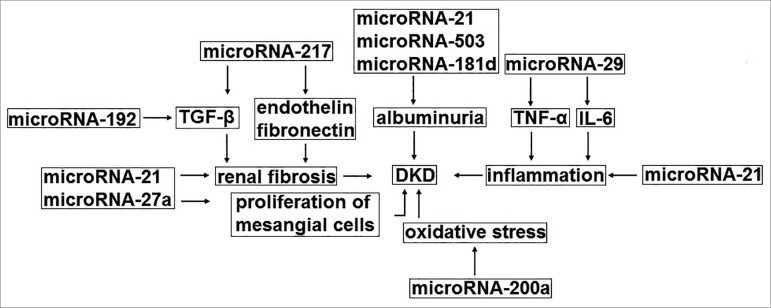
Mechanism of action of microRNAs with possible nephropathogenic properties. DKD = diabetic kidney disease; IL6 = interleukin 6; TGF-β = transforming growth factor beta; TNFα = tumor necrosis factor alpha.

Figures 1 and 2 describe the mechanism of action of microRNAs with possible nephroprotective or nephropathogenic properties. Stimulating the expression of microRNAs with possible nephroprotective effects and inhibiting the expression of microRNAs with possible nephropathogenic properties is a promising strategy for the treatment of DKD.

## Conclusions

Numerous microRNAs are involved in the pathogenesis of DKD by increasing the expression of molecules linked to inflammation, fibrosis, and oxidative stress. Therefore, they are promising therapeutic targets for DKD. Stimulating the expression of nephroprotective microRNAs may aid in the prevention and treatment of DKD.

Since the serum and urinary levels of different microRNAs change before increases in albuminuria and decreases in GFR are observed, and given that they are relatively stable in these biological materials, microRNAs may be relevant biomarkers for the early diagnosis of DKD. Additionally, the levels of some microRNAs change with the progression of DKD, thus possibly making them useful markers to monitor the progression of DKD.

Despite the limitations inherent to this study, microRNAs might become valuable additions to the list of biomarkers currently available for the early diagnosis and monitoring of DKD, in addition to serving as possible therapeutic targets for drugs developed to enhance the treatment of DKD.
